# Effect of Fillets on Mechanical Properties of Lattice Structures Fabricated Using Multi-Jet Fusion Technology

**DOI:** 10.3390/ma14092194

**Published:** 2021-04-24

**Authors:** Aamer Nazir, Ahmad-Bin Arshad, Chi-Pin Hsu, Jeng-Ywan Jeng

**Affiliations:** 1 Department of Mechanical Engineering, National Taiwan University of Science and Technology, 43 Keelung Road, Section 4, Taipei 10607, Taiwan; aamernazir.an@mail.ntust.edu.tw (A.N.); ahmed9c23@gmail.com (A.-B.A.); 2 High Speed 3D Printing Research Center, National Taiwan University of Science and Technology, No. 43, Section 4, Keelung Road, Taipei 10607, Taiwan; bingohsu@mail.ntust.edu.tw; 3 Graduate Institute of Biomedical Engineering, National Taiwan University of Science and Technology, No. 43, Section 4, Keelung Road, Taipei 10607, Taiwan; 4 President Office, Lunghwa University of Science and Technology, No.300, Sec.1, Wanshou Rd. Guishan District, Taoyuan City 333326, Taiwan

**Keywords:** additive manufacturing, lattice structure, design for AM, unit cell, fillets, energy absorption, loading–unloading

## Abstract

Cellular structures with tailored topologies can be fabricated using additive manufacturing (AM) processes to obtain the desired global and local mechanical properties, such as stiffness and energy absorption. Lattice structures usually fail from the sharp edges owing to the high stress concentration and residual stress. Therefore, it is crucial to analyze the failure mechanism of lattice structures to improve the mechanical properties. In this study, several lattice topologies with fillets were designed, and the effects of the fillets on the stiffness, energy absorption, energy return, and energy loss of an open-cell lattice structure were investigated at a constant relative density. A recently developed high-speed AM multi-jet fusion technology was employed to fabricate lattice samples with two different unit cell sizes. Nonlinear simulations using ANSYS software were performed to investigate the mechanical properties of the samples. Experimental compression and loading–unloading tests were conducted to validate the simulation results. The results showed that the stiffness and energy absorption of the lattice structures can be improved significantly by the addition of fillets and/or vertical struts, which also influence other properties such as the failure mechanism and compliance. By adding the fillets, the failure location can be shifted from the sharp edges or joints to other regions of the lattice structure, as observed by comparing the failure mechanisms of type B and C structures with that of the type A structure (without fillets). The results of this study suggest that AM software designers should consider filleted corners when developing algorithms for generating various types of lattice structures automatically. Additionally, it was found that the accumulation of unsintered powder in the sharp corners of lattice geometries can also be minimized by the addition of fillets to convert the sharp corners to curved edges.

## 1. Introduction

Cellular structures with multifunctional properties are common in nature and have shown promise for applications in the automotive, biomedical, and aerospace industries. Studies have revealed that mechanical properties, such as the strength-to-weight ratio [1,2,3,4], stiffness [5], acoustic [6] and thermal properties [7], energy absorption [8,9,10], electrical conductivity [11], and impact resistance [12], can be simultaneously improved by tailoring the topology of the cellular structures. Recent developments in additive manufacturing (AM) technologies, such as improved accuracy, higher fabrication speed, and the use of advanced materials, make it possible to fabricate cellular structures with intricate architectures, thus enabling the investigation of unexplored characteristics of these structures. By using AM techniques, cellular structures with uniform, and/or nonuniform microstructures can be constructed to obtain the desired properties for a particular application [13,14]. AM techniques have many advantages over traditional manufacturing processes, including quicker and less costly manufacturing, no tooling or material waste, the capacity for customization and personalization, and the ability to manufacture complex geometries, such as cellular structures, without additional cost or difficulty [15].

Although cellular structures have many excellent characteristics, they cannot be fabricated at the production level without using a high-speed AM system that can manufacture complex geometries with high accuracy and the desired functional properties [16]. Therefore, a recently developed high-speed AM technology called multi-jet fusion (MJF) was used in this study [17].

An important characteristic of cellular structures is the ability to accommodate large deformations through material and/or topological nonlinearities [18,19]. This property has been exploited in protective devices, such as protective military and sports equipment [20], crashworthy vehicles, shoe midsoles [21], reusable energy absorption devices, truss-like energy-absorbing medical implants [22], soft robotic actuators and structures, having negative Poisson ratio effects [23,24]. During an energy-absorbing large deformation process, these structures tend to decrease the maximum stress transferred through the material by evenly distributing the stress until the structure is densified [25]. 

Several studies have investigated the energy absorption capacity of cellular structures [26]. For instance, Fan et al. [27] studied the energy absorption behavior of tubular structures and revealed that using higher-order tubular cellular structures can improve the energy absorption efficiency. Al-Saedi et al. [28] used compressive testing to investigate the energy absorption behavior of functionally graded lattice structures fabricated using an aluminum alloy in a selective laser melting (SLM) system. Ozdemir et al. [29,30] used numerical and experimental methods under quasi-static and dynamic loading conditions to investigate the energy absorption behavior of re-entrant, diamond, and cubic lattice structures fabricated using AM techniques. Shen et al. [31] performed experiments at high strain rates to investigate the energy absorption and compressive behavior of closed-cell aluminum foams. A number of researchers have investigated the effect of high-performance unit cell shapes on the behavior of these structures under compression, deformation, and crushing. Ullah et al. [32] performed numerical and experimental studies to predict the energy absorption capacity of Kagome and atomic cell lattice structures unit cell fabricated by SLM. In another study, Li et al. [33] showed that mechanical properties such as the deformation, elastic modulus, and strength can be varied by changing the unit cell shape. Ashby [34] categorized cellular structures into two classes: stretch-dominated and bending-dominated structures. This concept is considered very useful for analyzing the mechanical properties of lattice structures. In a recent study, Kaur et al. [35] used experimental and simulation-based methods to investigate additively manufactured stretch-dominated lattice structures fabricated using polymeric materials. Ajdari et al. [36] performed a numerical study to investigate the effect of defects, irregularities, and deformation rate on the energy absorption behavior of honeycomb structures. However, they did not validate their numerical study. In 2018, Habib et al. [37] analyzed various open-cell lattice structures experimentally and using simulations to determine the optimal structure for energy absorption applications. They used the polymeric material nylon 12 to fabricate compressive samples using an MJF 3D printing system. However, they investigated only the energy absorption properties of some lattice topologies without analyzing the loading–unloading behavior of these structures. In another study, Zheng et al. conducted tension and compression testing of ultralight microarchitecture lattice structures with a constant stiffness per unit mass density and found that these structures are ultrastiff regardless of the type of material used to fabricate them.

The existing literature on lattice structures for energy absorption focuses mainly on 2D honeycomb structures [38], and few researchers have investigated 3D structures, because they have complex geometries and are difficult to model. The literature provides a basic foundation; however, deeper investigation is needed to answer various questions about high-performance lattice structures, e.g., which parameters should be modified to improve the energy absorption performance, how failure can be avoided, and how the stress concentration can be improved by modifying the unit cell of the lattice structure for specific applications. Addressing these concerns is the main purpose of this study. A literature review revealed that a number of researchers have investigated several high-performance lattice structures [39,40]; however, very few have modified a high-performance lattice structure to identify an optimal unit cell geometry with the maximum energy absorption and lowest stress concentration that exhibits stable failure. 

The literature reveals that very few researchers have studied the loading–unloading behavior of lattice structures, which is crucial for various industries, particularly the footwear industry, in which it can enable the design and fabrication of customized and personalized midsoles and insoles. Therefore, the loading–unloading behavior of a high-performance lattice structure and modified geometries of this structure is also investigated in this study. Furthermore, recent studies [41,42] on the buckling and postbuckling behavior of lattice structures revealed that the lattice unit cell fails from the corner owing to residual stresses at sharp corners, i.e., the lattice unit cell needs to be stronger at the corners to withstand high stresses. Therefore, modified structures with curved corners were designed to investigate the effect of curved corners on the performance and failure mechanism of the lattice structures in this study. Both experimental and simulation-based studies were performed to evaluate the performance of unit cells with Kelvin structure and simple or modified shapes.

In this study, several lattice topologies with fillets added to horizontal and vertical struts were designed to investigate the effects of the fillets on the stiffness, energy absorption, return, and energy loss of open-cell lattice structures with the same relative density. Furthermore, additional horizontal and vertical struts were added in a simple Kelvin structure to investigate the effect on stiffness and energy absorption. A recently developed high-speed AM MJF technology was employed to fabricate lattice samples with two different unit cell sizes. Uniaxial compressive experiments and nonlinear finite element analysis (FEA) were used to investigate the stiffness and failure mechanism. The energy absorption and loss were determined by performing loading–unloading experiments to obtain the energy absorption and energy return data, which were used to calculate the energy loss of each type of lattice structure.

## 2. Design and Fabrication Methods

The methodology includes the design of the lattice unit cells, their fabrication using an MJF 4200 3D printer (CA, USA), and sample testing on a universal testing machine.

### 2.1. Lattice Structure Design

**Figure 1:**
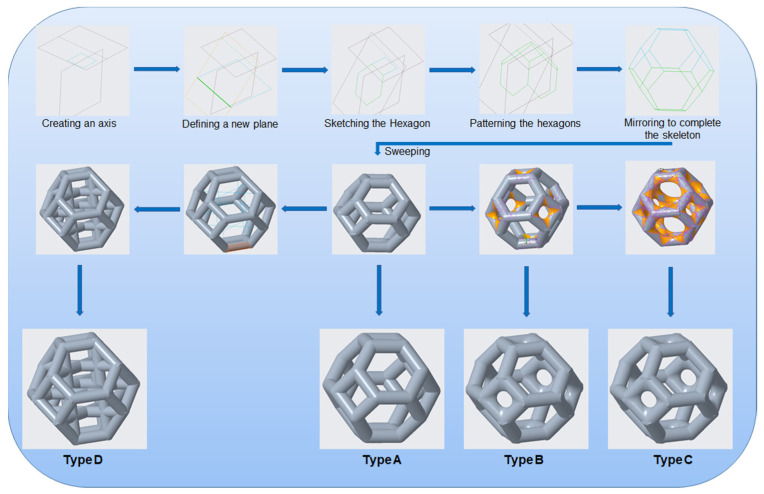
Methodology used to design the four types of lattice unit cell investigated in this study.

**Figure 2:**
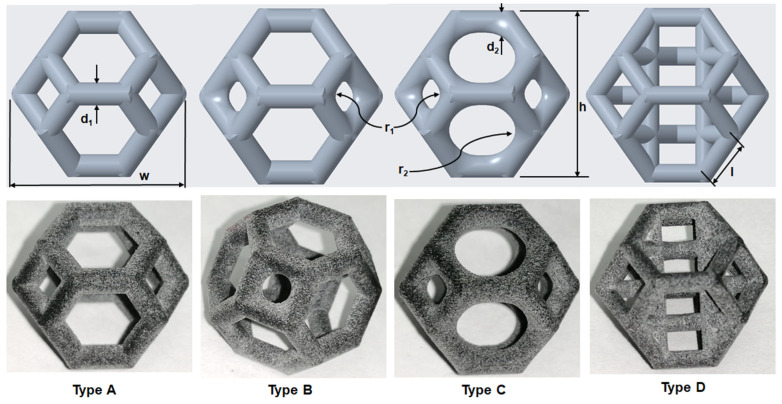
**Top**: front view of unit cell designs and design parameters to obtain the same relative density. **Bottom**: Lattice unit cell 3D-printed using polyamide 12 on an HP MFJ 4200 3D printer.

The mechanical properties of lattice structures are usually expressed in terms of the relative density, which is the most important property of cellular/porous structures and is defined as the ratio of the apparent density of the lattice structure to the density of the solid material from which the structure is constructed [43,44]. Therefore, the relative density was kept constant to compare the mechanical performance and energy absorption of lattices with a simple Kelvin unit cell and three modified Kelvin unit cells. The systematic design procedure is illustrated in Figure 1. These modified structures were designed to strengthen the cell edges, which ultimately reduces the residual stress and also changes the failure mechanism. For each type of lattice (Table 1), unit cells with dimensions of 10 × 10 × 10 mm^3^ or 20 × 20 × 20 mm^3^ and geometrical symmetry along the three principal axes were designed. Figure 2 and Table 1 show the morphology, design parameters and dimensions, unit cell size, and relative density of each type of lattice structure. The strut diameter is different for each type of lattice unit cell to keep the relative density constant. 

The design depends on the angle of the hexagonal faces with respect to the square faces. This plane has to be at a specific angle; otherwise, the edges of two adjacent hexagons will not be collinear. To define this plane, it is necessary to consider the hexagonal faces of the Kelvin structure as the continuation of the sides of an equilateral square pyramid (Johnson Solid J_1_). This plane can be identified by finding the height of the pyramid and drawing a line at the center of the base square (which is the top square face of the Kelvin structure) at that height, as shown in the upper left corner of Figure 1. This height is found as follows:

(1) $$h=\frac{1}{\sqrt{2}}l$$

where h is the height of the pyramid, and l is the edge length of the square face of the Kelvin structure. Next, a plane is created such that the line at the top and the edge of the base square are coplanar. The hexagonal face lies in this plane. A hexagon is drawn in this plane using the edge of the square at the top. This hexagon is repeated in a circle around the central axis to obtain the top half of the structure. The top half is mirrored to obtain the full Kelvin structure. Finally, the sweep command is used to form a solid body from these lines by specifying the shape and dimensions of the swept body of the type A unit cell. All the unit cells have struts with circular cross sections of diameter d_1_ and d_2_, as shown in Figure 2. The round command in the Creo software was used to remove the sharp edges at the ends of the circular sections. Unit cells of types B and C were designed by selecting the edges and using the round command to add material to the nodes. Types A, B, and C are bending-dominated structures because their deformation behavior is dominated by bending, whereas type D is buckling-dominated owing to the presence of vertical struts that fail by buckling. The unit cell with type D structure was designed using the sketch command to join the vertices of the opposite square faces. All the opposite square faces were joined in the same manner. Finally, the sweep command was used to create solid cylinders along the lines.

### 2.2. Fabrication of Lattice Structure

The recently developed HP MJF 4200 3D printer was used to fabricate the samples for all of the experiments, including the tensile, uniaxial compression, and loading–unloading tests. This printer can print parts with high accuracy, high functionality, and an excellent surface finish [16]. Additionally, the layer effect was not obvious on the surface of the printed parts, i.e., the printed samples were nearly isotropic and were not significantly affected by the build orientation. The working principle and procedure are described in [16], published in 2019. All the samples, including dogbone samples for material testing, were printed in the same batch. The samples were placed equidistant from the center, and all the specimens were placed on top of each other. These steps were taken to minimize the effect of the printing process on the fabricated samples. Figure 2 shows the good appearance and surface accuracy of the 3D-printed samples, which are superior to those of samples obtained using other AM technologies. 

## 3. Simulation Framework

### 3.1. Material Properties

**Figure 3:**
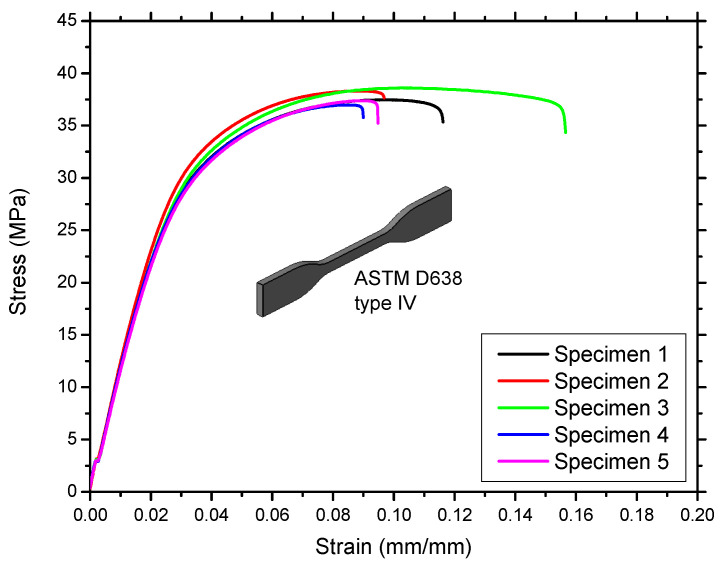
Tensile testing curves showing the nominal stress–strain behavior of PA12.

Five samples of tensile standard type IV were printed in the same batch as the lattice unit cells to determine the mechanical properties of polyamide 12 (PA12). These samples were tested in accordance with the ASTM D638 standard. An MTS universal testing machine (MTS Systems Corporation, Eden Prairie, MN, USA) with a 10 kN load cell was used to test the tensile specimens at room temperature by uniaxial tensile testing at a speed of 5 mm/min. The applied load and the corresponding deformation data were continuously recorded using TestWorks 4.0 software [45], which was integrated with the MTS testing machine. The nominal stress and corresponding strain percentage obtained from the tensile tests are shown in Figure 3, whereas the linear and nonlinear properties of PA12 are listed in Table 2 and Table 3, respectively.

The true stress and true plastic strain were calculated from the yield point to obtain the ultimate tensile strength (UTS) for use in the nonlinear FEA; the results are shown in Table 3. As shown in Figure 3, there is no significant data scattering in Young’s modulus, tensile strength, and ultimate strength measurements. The ultimate tensile strain values are much more scattered; however, this is not an issue in the present work since these experimental results will be used in simulations until yield point only.

### 3.2. Finite Element Analysis of Lattice Unit Cells

The static structural module available in ANSYS Workbench [47] was used to perform FEA of the cellular structure of the unit cells. The samples were imported to ANSYS as .step files from PTC Creo. The material properties, including the nonlinear stress–strain data, were defined in ANSYS. The linear and nonlinear properties in Table 2 and Table 3 were used as the material input parameters for FEA.

**Figure 4:**
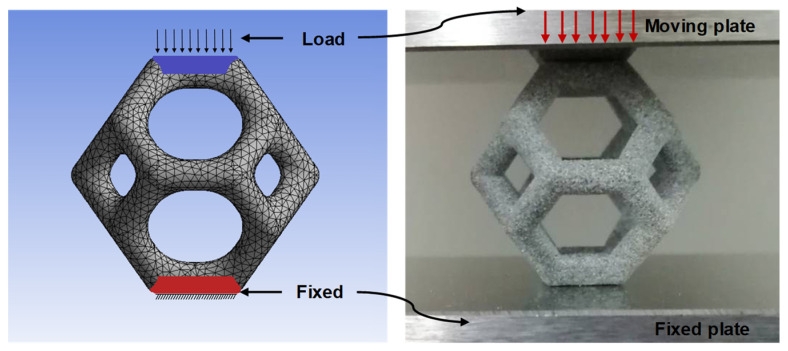
**Left**: FEA setup showing the mesh, load application, and boundary conditions. **Right**: Setup for uniaxial compression and loading–unloading tests.

To obtain more accurate simulation results, a mesh dependency study was first performed, and the mesh size was set to 0.4 mm for the Tet10 elements in the simulation of all lattice structures. The mesh convergence study revealed that meshes smaller than 0.4 mm cannot provide more accurate lattice structure results without an exponential increase in the computing cost. The load and boundary conditions are illustrated in Figure 4. A uniaxial compressive load was applied to the top surface and the bottom surface was fixed for all the axial displacements as well as transverse translations and rotations. Time-step control was turned on in the analysis settings; the minimum time step was 0.001 s, and the maximum time step was 0.1 s.

The behavior of the lattice structures above the elastic limit is very complex owing to various types of nonlinearities such as geometric, contact, and material nonlinearities. Consequently, postyielding simulation of the lattice structures is very challenging, and the results are very different from the experimental results. This problem is more severe for specific types of lattices that fail initially owing to local buckling of the struts, followed by stretch- and bending-dominated failure. Since various types of lattices including bending- and buckling-dominated lattices are studied here, some structures were simulated until the onset of failure, whereas the buckling-dominated structures were simulated only up to the elastic limit.

## 4. Experimental Results and Discussion

Uniaxial compression and loading–unloading tests were performed in accordance with the ASTM standard D1621-16 [48]. For each lattice type, three specimens for each unit cell size were tested at room temperature in an MTS universal testing system with displacement control. The data scattering on the measurement was limited to 4%. Based on three experiments of each design configuration, average results were calculated and are reported on the load-deformation curves in this section. Then, the average results for each design configuration were calculated from three tested specimens for each designed lattice unit cell. According to the ASTM standard, the test speed was set to 10% of the total height of the sample per minute. The test was stopped when the specimen was deformed to 50% strain.

### 4.1. Effect of Fillets on Lattice Structures 

**Figure 5:**
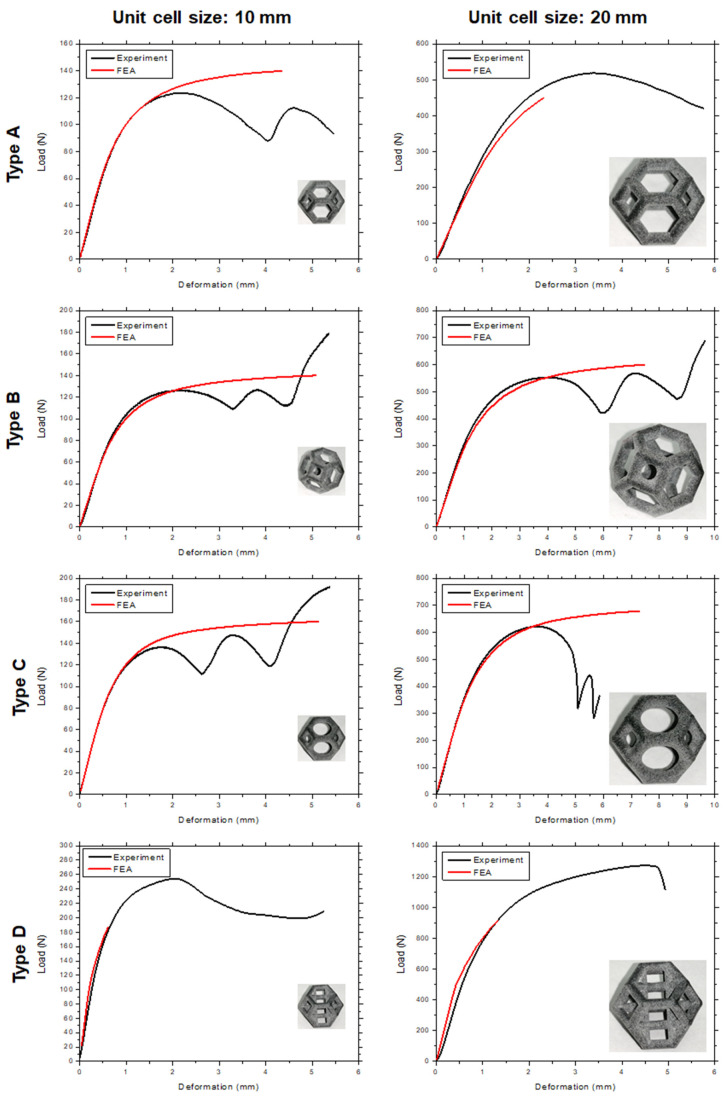
Experimental uniaxial load–displacement curves of 10 mm (**left**) and 20 mm (**right**) unit cells for all lattice morphologies.

**Figure 6:**
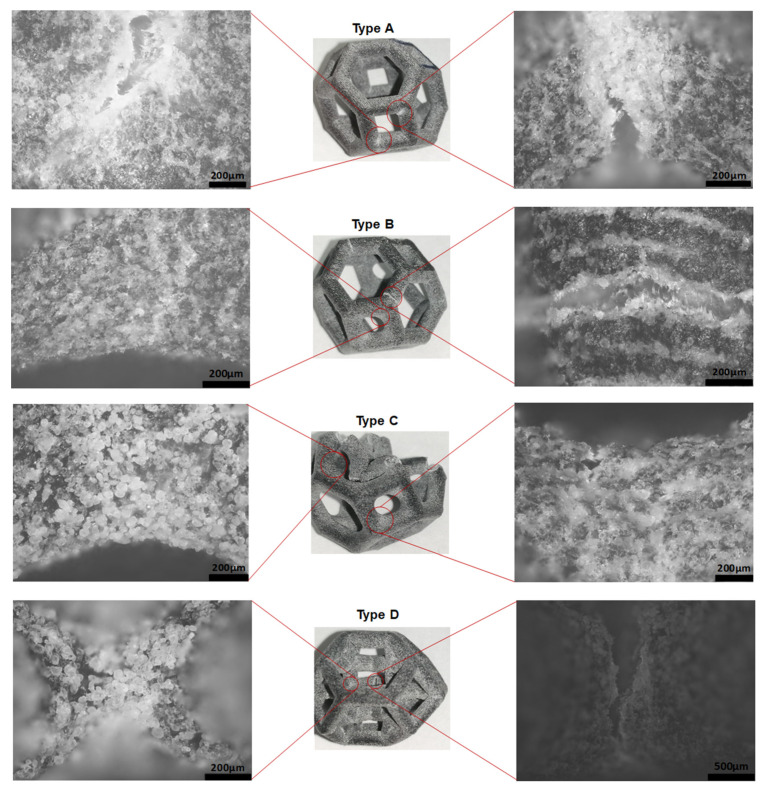
Micrographs showing the failure mechanism of the unit cells with and without fillets.

Figure 5 shows the load–deformation curves of 10 mm and 20 mm unit cells of each type of lattice structure with a constant relative density for each unit cell size. The type D structure clearly has the highest load-bearing capacity, followed by types C, B, and A, in that order. A similar trend was recorded for the unit cells of both sizes for all the lattice structure types. The modified structures (types C and B) have a higher stiffness than type A (without fillets) owing to the addition of fillets at the edges, i.e., more material was distributed at the edges, increasing the stress concentration. Since the type D structure had more vertical struts [41], the strength increased by 100%, compared with that of the type A structure. The vertical struts increased the buckling strength [40], allowing the type D structure to bear a larger load, which is reflected in the greater strength of the structure. Figure 6 shows micrographs of all the lattice structures, which clearly show the failure mechanism and the effect of fillet addition on the failure mechanism and failure location for each type of lattice morphology.

The type A structure failed (Figure 6) from the top and bottom vertices of the square faces parallel to the loading direction. This failure was due to bending in the region of the maximum residual stress because of the sharp edges. 

The addition of fillets at these square vertices in the type B structure shifted the failure point from the vertices of the squares to the beams extending upward from these square faces to form the hexagonal faces (Figure 6), and failure occurred closer to the end of the beams. The filleted edges of the squares were intact, showing no evidence of cracking. Additionally, the type B structure showed higher stiffness and ultimate compressive strength (Figure 5) than the type A structure; however, it also failed at a lower strain. Moreover, observations revealed that crack propagation was significantly slower in the type B structure than in the type A structure. 

Failure originated not from the squares but rather from the top struts, which cracked from the middle. The reason is that the top beam undergoes the most warpage and pitting. The struts above the top fillet have a high stress concentration, as indicated by white areas (Figure 6). It is shown that the failure has shifted from the squares to the hexagonal beams because the top struts crack from the middle and turn downward; this bends the following struts downward as well by the cantilever effect on these beams, which fail by bending. The micrographs also show that the cracks originated from the layer interfaces.

The failure mechanism of the type C structure differed greatly from those of all the other structures (Figure 6). The failure mechanism can be understood by considering the variable mass distribution in specific locations of the structure, i.e., more mass was taken from the middle of the struts and added to the edges of the structure to minimize the stress concentration. Therefore, the strut diameter in the middle of the struts in the type C structure is smaller than the strut diameter at the same position in the type A structure (Table 1), in which the relative density of the structures is the same. This reduction in the strut diameter in the middle of the strut makes the struts more prone to failure before the edges. In addition, warpage and/or cracks were observed in the struts that form the top and bottom square faces of the structure owing to the variable strut diameter. However, this structure has significantly higher stiffness and ultimate strength than its type A and type B counterparts.

**Figure 7:**
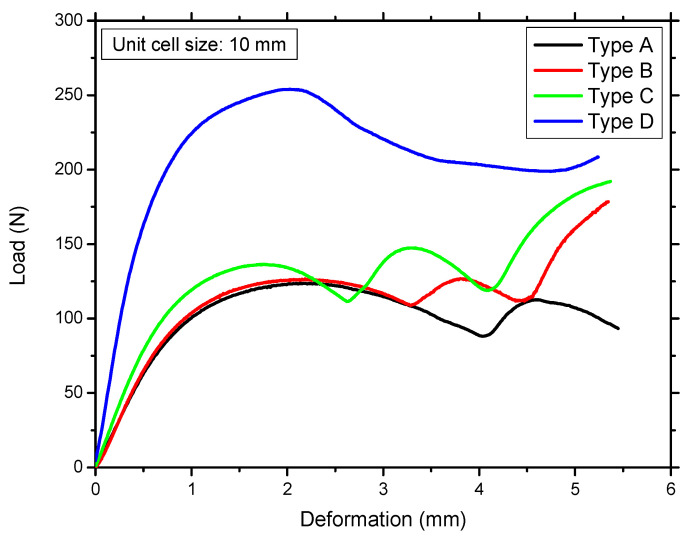
Experimental load versus displacement curves of all structures.

The failure mechanism and mechanical properties of the type D structure are very different from those of all the other structures discussed above. This structure showed significantly higher stiffness than all the others (Figure 7). Additionally, it failed at a much lower strain percentage than the others. It failed primarily by the buckling of the inner vertical struts, which absorbed a significant amount of energy before local buckling occurred. These vertical columns in type D were initially protected from buckling by the horizontal supporting struts, which were stressed in tension, whereas the other sections of the struts underwent compressive deformation. Buckling occurred when the vertical struts were compressed, and these struts applied a pulling force on the neighboring horizontal struts, pulling some of them in the direction of the buckling. This caused the horizontal struts to crack because of tension at their joints with the vertical columns. Since all the edges of the type D structure are sharp, failure occurs from the edges at which the vertical and horizontal struts join. 

**Figure 8:**
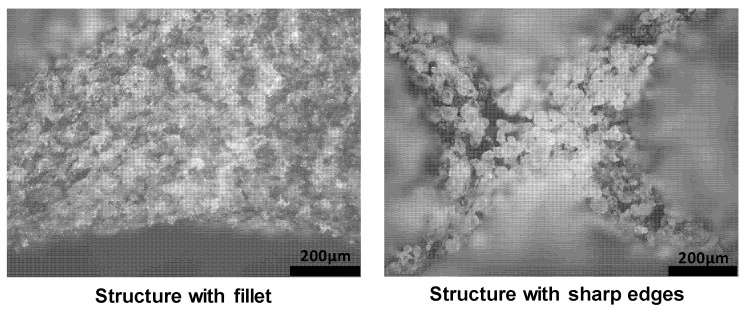
Accumulation of unsintered powder in the structure with sharp edges (**right**) and a structure with fillets (**left**).

The observations revealed that more unsintered powder accumulated in the structure that has sharp edges at which the struts join. By contrast, less unsintered powder adhered to the curved surface owing to the addition of fillets (Figure 8). 

In type A, B, and C structures (Figure 6), the failure mode is shifted from the joints to the beams by the addition of fillets. The structure with all filleted edges exhibited better stiffness and energy absorption (Figure 7). The structures became stiffer and had a higher ultimate strength; however, the filleted structures failed earlier because the strut was narrower at the middle of the beam. This finding indicates that a high-performance structure can be designed by optimizing the beam diameter, fillet radius, and shape of the structure for maximum energy absorption and strength-to-weight ratio. There are two methods of obtaining this optimal structure. One is to fix the fillets and change all of the strut diameters at the same time to design an isotropic structure. The second is to use an anisotropic design by shifting mass to where it is most needed. Optimization of the type D structure would be much more complex than optimization of the other structures. The variables for optimization would be the diameter of the outer struts, inner vertical struts, and horizontal struts and the presence of fillets with a suitable radius according to the unit cell size of the structure. 

### 4.2. Validation Using Simulation Results

The methods of performing the nonlinear simulation and determining the material properties are described in the simulation framework section. The multilinear isotropic hardening material model was used to define the nonlinear properties of the material. 

**Figure 9:**
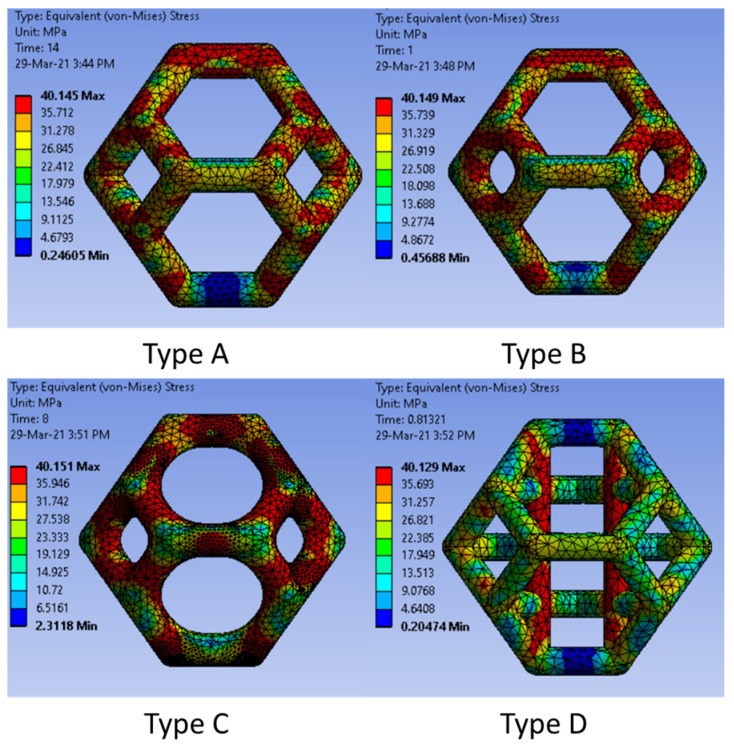
The von Mises stress distribution in all four types of unit cells studied in the present study.

Figure 5 compares the experimental and simulation results for the four lattice structures with two different unit cell sizes. The FEA and experimental data showed good agreement until the ultimate strength of each structure was exceeded. The struts broke after the stress in the beams exceeded the ultimate strength. In the FEA, the reason is that the material properties given to the simulation program are only those until the UTS is reached. The lattice structures exhibited very complex behavior after the UTS was reached owing to beam fracture, local buckling, densification after the UTS, etc. [43]; therefore, the behavior of the structures after the UTS will be simulated and thoroughly discussed in a separate study and is not within the scope of the present study. The FEA of the type D structure showed excellent agreement with the experimental results until the yield point was reached. The type D structure failed because of buckling, tension, and compression of various struts; therefore, it is challenging to simulate the failure because even after breaking, these members transfer the load by frictional contact with other struts. This type of simulation with self-contact can be simulated only using explicit solvers, which are cost-prohibitive for large systems. The differences between the experimental and FEA results may also be explained by geometric imperfections, the variability of the load and boundary conditions, variation in the material properties, and the variability of the beam thickness [49,50,51]. Figure 9 shows the equivalent stress distribution of the four types of Kelvin unit cells. In type A, maximum equivalent stress occurs on the nodes where the beams join each other. The beams themselves have a lower amount of stress, compared to the nodes. For types B and C, due to the addition of mass at nodes and a decrease in the beam diameter, the maximum equivalent stress is more uniformly distributed throughout the structure. In type D structure, the maximum stress occurs in the vertical beams and the nodes between vertical beams and inner horizontal beams. 

### 4.3. Effect of Fillets on Energy Absorption

**Figure 10:**
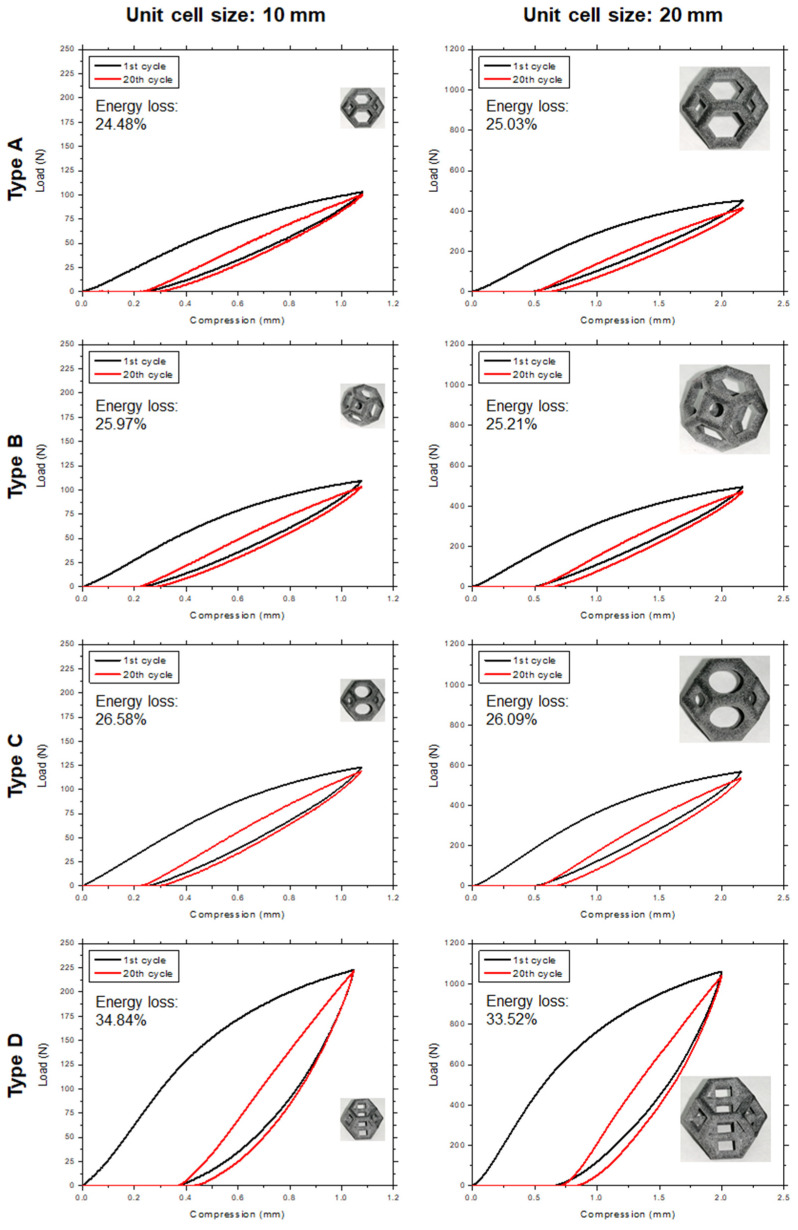
The 1rst and 20th loops of loading–unloading cycles showing the energy absorption, energy dissipation, and energy loss for each lattice type at two different unit cell sizes.

**Figure 11:**
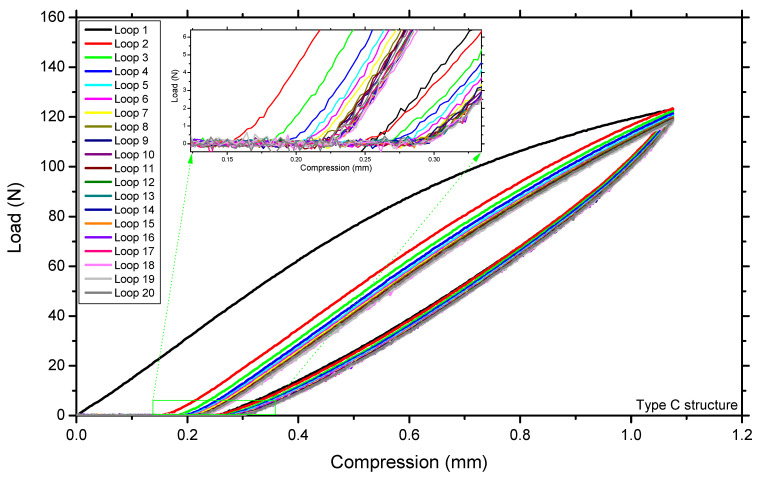
First 20 cycles of loading–unloading experiment.

Adding fillets to the lattice structure also had a significant effect on the energy absorption capacity of the lattice unit cells. To evaluate the energy absorption and resiliency properties of all the structures, loading–unloading experiments were conducted on the 3D-printed samples. Figure 10 shows the 1st and 20th loops of the loading–unloading cycles of the lattice structures with 10 mm and 20 mm unit cells. According to the literature, each sample needs to be compressed 20 times to obtain a steady-state hysteresis with a percentage change of only 3% [52]. Figure 11 shows the first 20 cycles of the loading–unloading experiment for the type C lattice structure. The energy absorption and return were calculated from the loading and unloading curves, respectively, by calculating the area under each curve (Figure 10). Equation (2) was used to determine the area under the loading and unloading curves of all the samples. The energy absorption and loss values for the 1st and 20th cycles for all the lattice types are listed in Table 4.

(2) $$A=\underset{n\to \infty }{\lim}\overset{n}{\underset{i=\infty }{\sum }}f\left(X_{i}\right)\Delta X$$

where f(X_i_) is the height, and ΔX denotes the width of the base of the small rectangle.

Figure 11 shows that the load–deformation curve for the first cycle starts at zero on the x-axis, whereas from the second cycle onward, the curves do not start at zero, i.e., permanent deformation occurred during the first cycle. However, we defined it as the material setting rather than permanent deformation. The maximum material setting was recorded for the type D structure, whereas the type C structure had the minimum material setting owing to the addition of fillets. Material setting occurred in the 3D printed components for many reasons; however, it is outside the scope of this study.

As shown in Figure 10 and Table 4, type A, B, and C structures show similar energy loss percentages for each cycle. The energy absorbed during loading is highest in the first cycle, and it decreases in each subsequent cycle until the fifth cycle, after which the differences between consecutive cycles are negligible. The energy released during unloading does not exhibit this trend but is almost constant from the 1st cycle to the 20th cycle. The energy absorbed and energy released both increase considerably from type A to the type C lattice. This increase is due to the addition of fillets, which stiffen the structure. At the same strain, the original Kelvin lattice (type A) structure can bear a 100 N load, whereas the type C structure (in which all the edges have fillets) can bear a 120 N load, which is 20% larger; however, the energy loss also increases. Owing to the increased stiffness of the structure, the compliance decreases, and each increase in stiffness results in the release of less energy or a higher energy loss percentage. 

The type D structure can absorb the most energy. The reason may be the presence of vertical members, which impart the most stiffness to this structure. However, the energy loss is also highest for the type D lattice. In addition, this structure can absorb slightly less than twice the energy absorbed by type A; however, its energy loss percentage is also significantly higher than those of the other types. Figure 10 shows that the structures with larger unit cells can absorb more energy, whereas the energy loss percentage is quite consistent, which implies that the loss percentage might be a function of the morphology and relative density of each lattice type. 

The maximum load at the same strain increases continuously from type A to type D. Type A, B, and C structures show similar values owing to their similar morphologies. Type A has the lowest stiffness and the lowest load at 10% strain. Type B and C structures have both higher stiffness and a larger maximum load. The type D structure has the highest stiffness and highest load among the structures. This characteristic is attributed to the presence of vertical beams, which are most resistant to axial compression. The presence of horizontal beams intersecting with the vertical beams increases the maximum buckling load of the vertical beams. The type D structure also has the greatest amount of material setting—at 3.5%.

**Figure 12:**
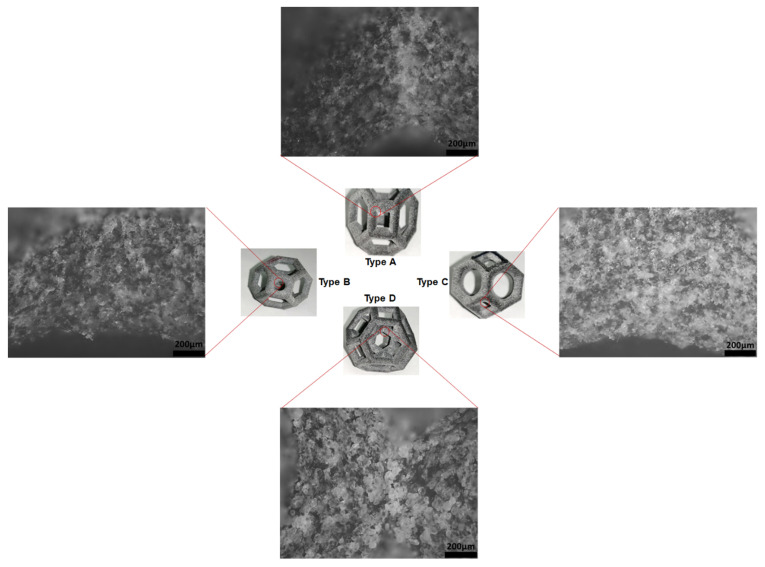
Micrographs of each type of lattice after loading–unloading tests in which they were compressed at 10% strain. There is no evidence of cracking; however, type A (without fillets) showed crack initiation and stress concentration.

Figure 12 shows all the lattice unit cells after loading at 10% strain, followed by decompression (unloading) to the original position. The micrographs in Figure 12 show no evidence of cracking or crack initiation in type B, C, and D structures; however, the type A structure shows some evidence of crack initiation and stress concentration. Type B and C structures show permanent deformation or material setting upon unloading, i.e., the circular regions of these structures become elliptical in shape (Figure 12). The addition of fillets mitigates these effects and strengthens the structure.

## 5. Conclusions

In this study, the effects of fillets and vertical struts on the stiffness, energy absorption, energy loss, and failure mechanism of the unit cells of open-cell lattices were investigated. Uniaxial compressive experiments and FEA were performed to investigate the stiffness and failure mechanism, and the energy absorption and loss were determined by performing loading–unloading experiments to obtain the energy absorption and energy return data, which were used to calculate the energy loss of each type of lattice structure. This study highlighted the importance of adding fillets to the sharp corners of different types of lattice structures to improve the mechanical properties and reduce the postprocessing and depowdering of printed parts.

The Kelvin lattice structure was found to absorb energy efficiently owing to its bending-dominated and compliant behavior; however, it has a lower modulus than stretch-dominated structures of the same relative density. The strength and stiffness of the Kelvin structure can be improved by adding fillets and/or vertical struts, which also influence other properties of the cell, such as the failure mode and compliance. An improvement of 20% in energy absorption was found in type C structure (all edges were filleted) when compared with type A (without fillets) structure counterpart. This trend was validated by investigating the energy absorption in two different sizes of unit cells. When fillets are added, the failure location can be shifted from the sharp edges or joints to other regions of the lattice structure, as observed by comparing the failure mechanism of type B and C structures to that of the type A structure (without fillets) at the same relative density. The results of this study suggest that AM software designers should consider filleted corners when developing algorithms for generating various types of lattice structures automatically. Postprocessing and depowdering of lattice structures are among the main challenges in AM research. It was found that the accumulation of unsintered powder in the sharp corners of the lattice geometry can also be minimized by the addition of fillets to change the sharp corners to curved edges. 

It is concluded that the addition of vertical struts to the structure can increase the stiffness; however, the resulting structure fails too early and in a brittle manner compared to structures without vertical struts. The energy absorption and loss are also influenced by the addition of fillets and vertical struts. Type B and C structures (with fillets) absorb more energy than the type A structure (with sharp edges). The literature contains only a few studies in this research area; therefore, further research is needed on the effects of the fillet radius on the stress concentration and energy absorption of lattice structures with various morphologies. Postprocessing and depowdering are even more important for closed-cell structures than for open-cell structures; thus, the effect of fillets on closed-cell structures should also be analyzed in future research.

## Figures and Tables

**Table 1:** Lattice unit cell shapes, design parameters, and relative densities of unit cells.

Type	Structure Name	Structure Shape	Unit Cell Parameters (mm)			Relative Density (%)	Bounding Box Dimension (cm^3^)
			Length (l)	Strut diameter (d)	Height (h)		
A	Simple Kelvin	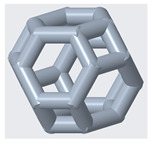	10.83	1.5	10.83	14.68	1.27
			21.66	3	21.66	14.68	10.17
B	Modified Kelvin with curved square corners	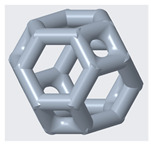	10.81	1.48	10.81	14.68	1.26
			21.64	2.97	21.64	14.76	10.13
C	Modified Kelvin with all curved corners	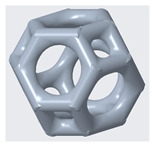	10.77	1.44	10.77	14.90	1.25
			21.55	2.88	21.55	14.91	10
D	Modified Kelvin with crossbars	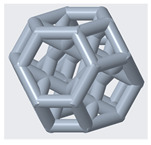	10.46	1.125	10.46	16.25	1.14
			20.92	2.25	20.92	16.25	9.15

**Table 2:** Material properties of PA12.

Density (g/cm^3^)	Young’s Modulus (MPa)	Poisson Ratio	Tensile Strength (MPa)	Ultimate Tensile Strength (MPa)
1.01	1150	0.33	23	38

**Table 3:** Nonlinear properties of the material for nonlinear FEA. Reprinted with permission from ref. [46] 2020, Springer Nature.

True Stress (MPa)	23.55	28.07	31.19	33.41	35.13	36.52	37.65	39.28	39.80	40.14
True plastic strain (mm/mm)	0	0.00625	0.01247	0.01865	0.02478	0.03088	0.03696	0.04898	0.05494	0.06085

**Table 4:** Energy absorption and loss for the 1st and 20th experimental cycles for all the lattice types.

Structure	Unit Cell Size (mm)	Energy Absorption		Energy Loss			
		1st Cycle	20th Cycle	1st cycle	%loss (1st Cycle)	20th Cycle	%loss (20th Cycle)
Type A	10	64.51	43.32	29.12	45.14	10.61	24.48
Type B	10	70.42	45.80	32.76	46.52	11.90	25.97
Type C	10	78.93	52.42	37.39	47.38	13.93	26.58
Type D	10	145.37	72.49	94.54	65.03	25.26	34.84
Type A	20	602.35	367.48	277.40	46.05	92.00	25.03
Type B	20	651.74	411.13	302.29	46.38	103.66	25.21
Type C	20	745.90	462.13	350.63	47.01	120.55	26.09
Type D	20	1348.19	664.19	850.74	63.10	222.64	33.52
